# Foreign Summary

**Published:** 1840-04-04

**Authors:** 


### FOREIGN SUMMARY.

*Four Cases of Aneurism of the Arch of the Aorta.** By JOHN REID, M. D., F. R. C. P. E., Lecturer on Physiology, President of the Anatomical Society, &c.—I have had occasion, within the last eighteen months, to inspect in the Royal Infirmary the bodies of nine individuals who died from aneurism within the thorax, and I have drawn up accounts of the morbid appearances observed. Four of these cases, from the rarity of their termination, appear to me to be of sufficient interest to be considered contributions to the morbid anatomy of aneurism of the arch of the aorta, and will be found in the following pages nearly in the same form, with the exception of a few verbal corrections, as I have entered them in the Registers of Dissections kept in the Infirmary. Of the five cases not detailed here, in one, the aneurism was placed upon the middle part of the thoracic portion of the aorta, and burst into the œsophagus; a second was placed upon the lower part of the thoracic and upper part of the abdominal aorta, and burst into the left cavity of the pleura; in a third, the aneurism was placed upon the transverse portion of the arch of the aorta, had destroyed by its pressure the continuity of the left recurrent nerve, and produced sudden suffocation by its effects upon the movements of the muscles attached to the arytenoid cartilages of the larynx; in a fourth, the aneurism occupied the upper part of the descending portion of the arch, and proved fatal by its pressure upon the trachea and left recurrent nerve; in the fifth, the aneurism was placed upon the trunk of the *arteria innominata* at its origin, had acquired an immense size, extending upwards as high as the thyroid cartilage of the larynx, and produced absorption of the upper part of the sternum, and of the inner end of the right clavicle. The skin had become livid at two places over the surface of the tumour, the blood had even begun to ooze out, but he died before any external rupture had occurred.

*Case* 1.—*Aneurism of the Aorta opening into the Right Auricle.*—E. W., aged 35, a tinsmith, and addicted to the use of ardent spirits, was admitted, 5th October, 1839, under the charge of Dr. Peebles. Stated that he had been long subject to fits of palpitation, but dates his present complaints from an uneasy sensation, like something giving way, which he felt in his chest while raising a heavy weight about three weeks before admission. On admission, the arms and chest, but more especially the face and neck, were of a livid colour, very much swollen, and pitted on pressure, and the vessels of the conjunctiva were congested with blood. The lower parts of the body were not œdematous.—He had much dyspnœa, and some cough. Strong impulse of heart, and its apex was felt lower than usual; and a double bellows sound was very audible over the cardiac and sternal regions, particularly over the upper part of the latter. The bruit accompanying the first sound of the heart was more prolonged; the bruit accompanying the second sound was sharper and shorter. The chest could not be satisfactorily percussed from the great œdema; but there was evident extended dulness over the cardiac region, and over the upper part of the sternum. The respiratory murmur was very loud at the upper angle of the scapula. He had some difficulty in deglutition; pulse 90, with a distinct interval between the impulse felt at the chest, and the beat at the wrist; urine scanty and turbid, not coagulable by heat or nitric acid. Had been previously bled, cupped, and had used diuretics and drastic purgatives. Both previous to and after his admission, his intellectual faculties were confused, and his speech was ultimately impaired. It appears, from information furnished by the medical gentlemen who attended him previous to his admission, that the lividity, œdema of upper part of body, and confusion of the intellectual faculties, occurred about the same time. On his admission the diagnosis was hypertrophy of the left side of heart, and aneurism of the aorta, and he was treated by drastic purgatives and digitalis. The œdema and lividity of upper part of body continued, and the dyspnœa became more urgent, and he was unable to assume the horizontal posture for any length of time. No œdema of lower part of body presented itself, except some slight swelling of scrotum. Some dark, circumscribed livid spots appeared on chest. He had delirium at night, and his eyes were bloodshot. Pulse ranged from 90 to 100. He died suddenly on the morning of the 13th.

*Sectio Cadaveris*, 16*th*.—The difficulty of prevailing upon the friends to permit an inspection of the body will explain its delay. Forty-four ounces of serum were effused into the cavity of the right pleura, and thirty six into the left. Lungs small, compressed by the serous effusion into the chest. They contained a considerable quantity of blood and frothy serum, but were everywhere spongy. The inner surfaces of the serous part of the pericardium were universally and firmly adherent, and the heart was decidedly larger than usual. The left ventricle was evidently somewhat dilated, while the other cavities of the heart were of their usual size. The walls of all the cavities of the heart were considerably thickened, as will appear from the

* Published by permission of the Managers of the Royal Infirmary, and of the Physicians under whose charge the patients were placed.

following measurement. Thickest part of right auricle 4-24ths, and thickest part of left 5-24ths of an inch; thickest part of right ventricle, 9-24ths, (measured where no *columnæ carneæ* were present;) thickest part of left ventricle, 18-24ths; thickness of middle part, 15-24ths; and of apex, 6-24ths. The walls of the heart were paler and more flabby than usual. The aortic and semilunar valves, and the other valves of the heart were healthy, and found adequate when tested by water. The aorta at its origin suddenly dilated, and formed an aneurismatic enlargement capable of easily containing the closed fist. This dilatation was confined to that part of the artery immediately above the aortic opening, termed the sinuses of Valsalva, and as the circumference was placed on a lower plane than the centre, the aortic opening, with its semilunar valves, was situated on the most elevated part of the floor of the aneurismatic swelling. The tendinous portions of the aortic opening were very much thickened, and somewhat elongated upwards; but the aortic orifice was not enlarged. The fibres of the middle coat of the artery which pass between the three projecting extremities of the tendinous festoons, and form the upper termination of the sinuses of Valsalva, were much elongated, and carried considerably upwards at the middle part, so that the upper boundary of the aneurism was bounded by three large segments of a circle, formed by these three bands of fibres. The ascending portion of the arch of the aorta suddenly resumed its original size, and there was a portion of the vessel 1 inch and 4-12ths in length, between the upper edge of the dilated part, and the origin of the *arteria innominata*, of its usual size and appearance, with the exception of being somewhat thickened and roughened on the inner surface. The aneurismatic dilatation chiefly projected backwards and to the right side, pressing upon the anterior edge of the *septum auricularum*, and the neighbouring parts of both auricles, and overlapping and pressing upon the anterior and left part of the right auricle, and stretching the termination of the descending cava over its outer surface. On examining the interior of the auricles and the aneurism, two oval openings, with defined and rounded margins, (one of which was 10-24ths of an inch in length, and 5-24ths in breadth; the other 9-24ths in length, and 5-24ths in breadth,) were observed forming a free communication between the aneurism, the right auricle, and the termination of the superior cava. The cava itself was pervious, but must have been compressed by the aneurism when distended with blood. On the inner surface of the anterior wall of the left auricle, near the anterior edge of the septum, there was a dark projecting part about the size of the point of the finger, which, from the pressure of the aneurism upon it, and the thinning of the walls of the auricle at that part, would soon have given way, and have formed a communication between the left auricle and the aneurism. On examining the inner surface of the aneurism, it was observed that the dilated coats of the vessel were thin and very soft, that they were deficient at some points, and that the sac of the aneurism was there formed by the adherent pericardium, or by the outer surface of the walls of the auricles. The blood was fluid, and there were no fibrinous clots in the aneurism. Abdominal organs healthy. Brain not permitted to be examined.

The old and firm adhesions between the inner surfaces of the serous part of the pericardium satisfactorily explains the unusual size of the aneurism in this patient. It is well known that, when an aneurism forms upon an artery, the internal and middle coats in general soon give way, and the aneurismal sac is then formed by the surrounding cellular tissue. When an artery is surrounded by very little cellular tissue, as is the case with that portion of the aorta enclosed within the pericardium, and with the arteries within the cranium, aneurisms formed upon it seldom acquire a large size; and there can be no doubt, from the appearances observed on dissection in this patient, that the aneurism would have burst into the cavity of the pericardium, and have soon arrested the heart's action at some period previous to the time of his death, and probably before it had burst into the auricle, had it not been for the old adhesions between the inner surfaces of the serous part of the pericardium. The greater thinness of the middle coat, and the larger calibre of the artery at the sinuses of Valsalva, account, as we have elsewhere explained,* for the greater liability of that part of the vessel to be affected with aneurism.

The sensation of something giving way in the chest felt by the patient on lifting a heavy weight, and the train of symptoms which followed, may be referred to some sudden increase of the size of the aneurism; and it is probable that at this time, also, the aneurism burst into the right auricle. The compression of the *cava superior* at its termination, satisfactorily explains some of the most striking symptoms of this case. It explains the great œdema and lividity of the upper part of the body, the congested state of the vessels of the conjunctiva; and, conjoined with the dyspnœa, will also account for the disturbance of the intellectual faculties. It also explains in a very satisfactory manner the effusion of serum into the chest, when none was effused into the abdomen; for as the *vena azygos*, which returns the blood from the inner surface of the parietes of the chest, enters the *cava superior* above the point compressed, the same impediment must have existed to the return of the blood from that vein, as from the other branches of the

* Article Heart, Cyclopædia of Anatomy and Physiology, Vol. ii.

*cava superior.* If the compression of the *cava superior* had occurred in a more gradual manner, as probably took place in a case of complete obliteration of this vein, which we have elsewhere detailed,* it is possible that the collateral circulation with the *cava inferior* might have been established without any such derangement of the circulation of the upper part of the body having manifested itself. Though all cases of compression of the *cava superior* are not attended by the same effects upon the circulation, yet at the same time it must, we think, be admitted, that, if similar marked symptoms of retardation of the blood in the vessels of the head and superior extremities with œdema, and the effusion of fluid into both sides of the chest, while the lower parts of the body remained unaffected, were presenting themselves, we would be justified in predicting that the *cava superior* would be found compressed.

Case 2.—*Aneurism of the Aorta bursting into the Pulmonary Artery.*—J. H. aged 36, of intemperate habits, was admitted on the 22d of May, 1839, into the Royal Infirmary, under the charge of Professor Alison. Stated that in January last, he began to feel occasional palpitations with dyspnœa, and underwent some medical treatment without relief. About a fortnight before admission, œdema of feet had commenced, and had continued to increase. On admission, the lips and face were pale. Had severe cough, and considerable dyspnœa, and much œdematous swelling of the feet and ankles; and also much enlargement of abdomen. There was considerable dulness on percussion over the cardiac region, and also over the lower part of the left scapula. The heart's action was irregular in force, and a bruit accompanied both sounds, but particularly the second, which was prolonged. The respiration was rather faint in the lower part of both sides of the chest, and was accompanied with subcrepitating râle; pulse small and weak; has occasional vomiting and sense of constriction in the epigastric region; micturition scanty and frequent; urine coagulable by heat and nitric acid; bowels costive. Took diuretics and antispasmodics without much relief to the symptoms. On the 5th June, it is reported that he feels, occasionally, as if he were going to faint; and has sense of uneasiness in the epigastrium; urine, from nineteen to twenty ounces daily. 19th. More dyspnœa last night; œdema of scrotum increased; cough continues; urine scanty. To take gtt. i. of croton oil, and two grains of hyoscyamus to be repeated. 24th. Has had drastic purgatives, (elaterine and gamboge,) which produced several watery stools, which were followed by a considerable diminution of œdema of scrotum and legs. 25th. A short time after noon, the dyspnœa became suddenly much increased, the lips became livid, the pulse feeble, and he died during the night.

*Sectio Cadaveris.*—Eighteen ounces of serum in the cavity of the abdomen; fifty-two ounces in the two sides of chest; and eight ounces in the cavity of the pericardium; heart considerably increased in size; right side of heart full of blood, chiefly fluid; right auricle considerably distended, without diminution of the thickness of its walls; right ventricle rather larger than usual; its walls somewhat increased in thickness, and much firmer than usual; left ventricle considerably dilated, without any increased thickness of its walls; mitral valve healthy. Two of the semilunar valves were elongated, and when thrown inwards, projected downwards into the upper part of the ventricle, and thus lay on a lower plane than the third valve, rendering them inadequate when tested by water. On the posterior and left side of the aorta, immediately above the semilunar valve, there was an aneurismatic swelling about the size of an orange, which projected backwards, and pressed upon the left auricle, and also partly lay on the right side of the aorta. On slitting up the pulmonary artery, a ragged transverse fissure was observed in the coats of the pulmonary artery 1 inch and 3-10ths in length, and commencing 1½ inch above the origin of the vessel from the right ventricle. This fissure formed a communication between the aneurismatic sac and the pulmonary artery. The coats of the aorta were thickened and irregular on the inner surface. Liver rather larger than usual, and granulated on the surface; and the cut surfaces presented a highly mottled appearance. The kidneys were slightly mottled by yellow deposit in the cortical portion; stomach much congested in the splenic extremity. Brain healthy.

From the appearance of the communication between the aneurism and the pulmonary artery in this case forming a narrow ragged fissure, it is probable that the pulmonary artery had given way a few hours before death; and at this time the dyspnœa had become suddenly increased with augmented lividity of the face.

Case 3.—*Aneurism of the Aorta communicating with the Pulmonary Artery.*—G. F. aged 60, was brought in a moribund state into the Clinical Ward under the charge of Professor Graham, aud died shortly after admission. No accurate account of the previous history of this patient could be obtained; but it appears that he had been seen by a physician in his own house, a month before he was brought to the infirmary; and he then evidently laboured under hypertrophy of the left side of heart, and had considerable dyspnœa.

*Sectio Cadaveris 36 hours after death.*—Some serous effusion in the cavity of the paricardium. The right side of the heart was distended with clots of blood, chiefly decolorized. The cavity of the left ventricle was con-

* No. 122 of this Journal.

siderably enlarged, with hypertrophy of its walls, and its apex projected an inch at least beyond that of the right ventricle. The lips of the mitral valve contained some thickened and hard spots at their fixed margins; but they were not shortened, and the auriculo-ventricular opening was not dilated. Several soft irregular warty excrescences adhered to part of the inner surface of the posterior and left semilunar aortic valves, and prevented their close approximation, though the valves themselves were not shortened. A few small warty excrescences were also attached to the aortic tendinous ring, immediately below the fixed margins of the semilunar valves. The ascending portion of the arch of the aorta was somewhat dilated, and presented numerous yellowish and slightly elevated spots on its inner surface. The part of the aorta from which the brachio-cephalic arteries arose, was nearly of its usual size, while it became suddenly dilated to about three times its usual calibre immediately beyond the origin of the left subclavian, and again assumed its natural size about an inch above the point where it passes behind the diaphragm. From the upper and right side of this dilated part of the descending portion of the arch, a small infundibuliform aneurismatic pouch, rather more than an inch in length, projected forwards and to the right side, and connected itself to the left branch of the pulmonary artery, about a quarter of an inch beyond the bifurcation of the pulmonary artery, into its left and right branches, so as to form a communication between the upper part of the dilatation and the left pulmonary artery. This infundibuliform aneurismatic pouch, at the part where it communicated with the dilated portion of the aorta, would have contained the point of the thumb, while the part which opened into the left pulmonary artery was not larger than the carotid or subclavian. The orifice of the opening into the left pulmonary was somewhat rounded, but at the same time irregular and fringed at the edges. The inner surface of the pulmonary artery presented several yellow spots in the immediate neighbourhood of this opening; and there was a small number of similar spots, but more scattered, placed a little above the pulmonary semilunar valves. At the upper part of the dilated portion of the descending aorta, the coats of the vessels were but little altered from their healthy appearance; but there were several yellow and calcareous deposits on the inner surface of the middle part. The lower lobes of both lungs presented each a circumscribed dense portion somewhat less than a pigeon's egg, (pulmonary apoplexy :) they were of a dark-red colour, and one of them contained a little puriform matter in its centre. Another dense portion nearly of the same size as those just described was found in the upper lobe of left lung, and was of a light brownish yellow colour. The liver was very pale, and somewhat resembled the fatty liver in its appearance; slight yellow granular deposit in the cortical structure of the kidneys. The spleen was larger and considerably firmer than usual.

The communication between the aorta and pulmonary artery in this case, exactly occupied the position of the *ductus arteriosus* in the fœtus. We believe, however, that the communication did not arise from the *ductus arteriosus* continuing pervious, from the circumstance that there was an aneurismatic dilatation of the aorta present at the point of communication; and the opening into the pulmonary artery was irregular and ragged, and did not present the smooth and rounded orifice of one artery branching off from the trunk of another. It may be difficult to decide whether this communication was or was not formed by the *ductus arteriosus* opened up, and somewhat altered by the presence of an aneurismatic dilatation on the aorta; but there can be no doubt that it lay exactly in the position of the impervious cord left in the adult by the obliteration of that vessel.

The parts exhibiting the three aneurisms of the aorta, and the parts into which they burst, detailed in the previous cases, as well as another case, which also occnrred in the Infirmary, of an aneurism of the aorta communicating with the pulmonary artery by two pretty large openings, related by Dr. Henderson in No. 127 of this Journal, are preserved in the University Anatomical Museum.

*Case* 4.—*Aneurism of the Aorta bursting externally.*—J. P. aged 31, a mason, of temperate habits, was admitted, 7th August, 1839, under the charge of Dr. Shortt. Stated that, about six years ago, he began to complain of pain behind the upper part of sternum; that about twelve months after this, a tumour about the size of a nut presented itself near the sternal end of right clavicle ; and that he has been occasionally troubled with dyspnœa, but not with palpitation. On admission there was a pulsating tumour, rising and falling with the systole and diastole of the heart, placed at the junction of the right clavicle with the sternum. The tumour was of a pyriform shape, projected about three inches in front of the sternum, and was about three inches and a-half in circumference. There was a loud bruit heard over the tumour, and a bruit also accompanied the sounds of the heart. Impulse of the heart strong, and felt below the sixth rib; complained of some pain in the region of the tumour. Of an athletic form of body, and general health good. Pulse 72, full and throbbing; no dyspnœa when quiescent, and appetite good; was ordered to be bled, and to take digitalis and colchicum. The tumour continued to increase in circumference, and there projected from its anterior surface a smaller tumour, measuring about three inches in circumference, and was prolonged outwards two inches beyond the surface of the broader tumour placed below. The pulsation was less strongly felt in the projecting portion than in the broad part of the tumour, but the skin covering the apex of it became first livid, and then of a dark-colour, and presented the appearance of incipient sloughing. On the 3d of September some oozing of blood took place from the apex of the projecting part. On the following day about two ounces escaped; and on the afternoon of the 4th, a sudden rush of arterial blood took place when he was straining at stool, and he soon ceased to breathe. The heart continued to beat for a few minutes after the respiration had ceased, and about four pounds of blood (to judge by the eye) had escaped.

*Sectio Cadaveris*, *6th*.—On opening the thorax, a large aneurismatic dilatation was found in the anterior mediastinum. This dilatation was formed by the aorta, and included all that part of the vessel placed between its origin from the heart, and where it lies on the fourth vertebra. The most dilated part was near the origin of the *arteria innominata*, and would readily have contained two fists. The walls of this dilatation were every where formed by the coats of the artery, except at the part behind and to the right side of the sternum. The middle coat of the artery was much thickened, and contained several calcareous plates at different parts. There was a rounded opening in the walls of the sac, which passed partly through a notch in the upper and right side of the sternum, and partly through the interval between the sternum, first rib, and clavicle. This opening admitted the passage of three fingers, and communicated with the aneurismatic tumour, which had appeared externally. The walls of the external tumour were formed by the anterior surface of the sternum, which was bare and rough, by cellular tissue, and by the skin. A firm fibrinous and decolorized clot, about a quarter of an inch in thickness, occupied the apex of the external tumour, and by the edges of this the blood had escaped. The internal tumour was partly occupied by a large soft dark-coloured coagulum. The left ventricle of the heart was very considerably dilated and hypertrophied. The semilunar aortic valves were shortened, thickened along their margins, and were inadequate. Lungs contained some serum, and were not particularly pale, some coagulated blood in heart. Liver and intestines pale; kidneys slightly granular.

This aneurism, though large, produced no dyspnœa under moderate exertion, and no dysphagia, from the circumstance that it projected forwards and not backwards. It is much rarer for aneurisms of the aorta to burst externally than what we would at first imagine. Some of the most experienced practitioners in this city have not witnessed such a case.

*Ed. Med. and Surg. Journ. Jan.*, 1840.

*Painful Cicatrix with Exostosis of the Metatarsal Bone of the great Toe.*

*St. Bartholomew's Hospital, Sept.* 1839.—Mr. Stanley removed the great toe with its metatarsal bone, from a man aged 30, for a disease of which the following is a brief history. Three years ago, while working on a railroad, he received an injury by which all the toes, except the great toe, were torn away from the left foot, and the soft parts on the inner side of the right foot were severely lacerated. Part of the cicatrix which remained after the healing of the laceration of the right foot, was seated immediately beneath the head of the metatarsal bone of the great toe, so that it was constantly exposed to the chief pressure in walking. The consequence was, that an ulcer formed upon the cicatrix, and though often healed, and apparently soundly, it as often broke out again when he endeavoured to take exercise. After several such alternations of healing and returns of the ulceration, the parts had become so excessively tender that even when the sore was cicatrized he was unable to bear any pressure upon the foot. The parts around the cicatrix also enlarged; the swelling extending towards the sole, and appearing to proceed chiefly from the head of the metatarsal bone of the great toe. After remaining for a considerable time in the hospital without any prospect of being by any other means relieved from the pain of the cicatrix, and the constant liability to fresh ulceration, the man consented to the removal of the toe and its metatarsal bone. The operation was accordingly performed, the incisions being so directed that the cicatrix that would follow might be placed as much as possible towards the upper surface of the foot, where it would receive the least pressure in walking. The wound healed soundly, and the man a short time after left the hospital, walking well and firmly, and without any prospect of future inconvenience.

On examining the parts after their removal, the chief bulk of the swelling was found to consist of a growth of osseous matter, of a firm cancellous texture, from the distal head of the metatarsal bone, just below and to the inner side of its articulating surface. The surface of the osseous growth was expanded, flattened, and rough; the surface of the adjacent part of the bone was also irregular, and the soft tissues, including the painful cicatrix, which was situated directly beneath the exostosis, were firmly adherent to it. The cartilage was removed from the articular surface of the metatarsal bone, and the phalanx of the toe had been thrust upwards by the tumour, so that it was nearly dislocated, and its axis formed nearly a right angle with that of the metatarsal bone.

*Unusual Arrangement of Parts observed in the Dissecting Rooms.*

1. The left subclavian artery, in the body of a male subject, passes in front of the scalenus anticus muscle. The right subclavian has its usual relations, and each gives off its usual branches; but the right vertebral artery is considerably smaller than the left. The other main arteries have their usual arrangement.

2. In a female subject the right vertebral artery is given off from the right carotid, which is the first great artery that arises from the arch of the aorta. Next to it arises the left carotid, which is so near to it that they almost appear to have a common origin. Next to the left carotid arises the left subclavian artery, which pursues its usual course and gives off its usual branches; and behind, and a little to the left of it, arises the right subclavian, which, coming off from the back of the arch of the aorta, passes to the right and upwards, crossing behind the œsophagus towards the first rib, over which it passes behind the scalenus anticus, giving off in its way all its usual branches except the vertebral, which is supplied by the right common carotid. This right vertebral is smaller than the left; it is given off by the carotid rather more than an inch from its origin, and like the left, passes into the foramen of the transverse process of the sixth cervical vertebra.

3. In the same subject there is a peculiar arrangement of the tendons about the shoulder-joints. On each side the long tendon of the biceps flexor cubiti divides, just below the level of the tuberosities of the humerus, into two portions, of which one, the smaller, is attached to the bottom of the bicipital groove between the tuberosities, and the other, the larger passes over the joint on the outside of the capsular ligament, to be attached to the upper edge of the glenoid cavity. The tendon of the pectoralis minor on each side, instead of being attached to the coracoid process, divides into two portions, which pass through an aperture in the coraco-acromial ligament, and of which one is lost on the upper and outer part of the fibrous capsule of the joint, near its attachment to the neck of the humerus, while the fibres of the other are continued to the upper margin of the glenoid cavity.

4. The arrangement of the tendons of the bicipites, in this case, is similar to that described by Mr. Stanley, in the lectures at the College of Surgeons, in which he showed that this is a permanent condition of that structure, which is normal in the early embryo. It is probable that in many of those cases in which the tendon of the biceps appears to be attached to the bicipital groove, and which are commonly supposed to be the consequence of a rupture of that tendon, it really passes in a thin band over the outside of the joint to its usual attachment, on the edge of the glenoid cavity. Such cases are of course to be regarded as original malformations, rather than as the results of injury or disease, of which they rarely present any coincident effect.

*Perforating Ulcer of the Stomach.*

*Nov. 22d.*—The man who was the subject of this disease walked up to London a fortnight ago, from a distance of two hundred miles, and had since that time been engaged in very laborious work, without any apparent inconvenience. He was extremely robust and active, and was not habitually intemperate. Last evening, at 9 o'clock, he was suddenly seized with the most acute pain in the abdomen, which was most intense around the umbilicus, and was somewhat increased by pressure. A respectable surgeon, who was immediately called in, administered some aperient medicines and other means, but the patient continued to sink rapidly during the night, and died at ten o'clock this morning, as he was being carried into one of the wards.

At the post-mortem examination, the abdominal cavity was found to contain a considerable quantity of air, and about two quarts of dirty yellowish fluid, with castor oil floating on its surface. The intestines were streaked with red, and very vascular on their peritoneal surfaces, and their walls were slightly adherent together by soft recently effused lymph. This peritonitis was found to have been caused by the effusion of the contents of the stomach through an ulcerated aperture in its walls. The ulcer was of an oval form, about half an inch in diameter, with sharp abrupt edges, as if it had been cut out. It was situated about an inch to the right of the œsophageal opening, just in front of the attachment of the gastro-hepatic omentum; the cellular tissue, for some distance around it, was thickened and brawny, and slightly puckered, like that of a cicatrix. There was another ulcer very near the preceding, of rather less size, but of the same form and characters, which had also penetrated all the coats of the stomach, effusion being prevented only by the adhesion of its edges to the anterior surface of the pancreas. In every other respect the man's body presented the appearances of perfect and very robust health.

A case in many respects similar to this occurred in the course of the summer. A young woman who up the moment of the commencement of her severe illness had considered herself in perfect health, and who was very robust and muscular, was suddenly seized while in bed with most acute pain in the abdomen, aud other symptoms of severe peritonitis. Various means were employed without the least affect, and she was brought in a dying state to the hospital, where she soon expired, twenty hours from the first seizure. With all the appearances of acute peritonitis there were found two ulcers of the stomach, about half an inch wide, of a circular or oval form, with hard, sharp, and abrupt edges. One of them had completely perforated all the membranes of the stomach, and had permitted the escape of its contents through a wide aperture; the other had also penetrated all the membranes, but it was closed behind by its adhesions to the pancreas. Both these ulcers were situated near to the œsophageal opening, and the tissues around them were thickened and dense.

Cases similar to these are recorded by Dr. Abercrombie, who particularly remarks on the singularity of their occurrence, in persons who appeared just previously to enjoy perfect health. They are also of some interest, from the suspicion which they may excite, as they did in both these cases, that the patients had died by some unfair means. Such a suspicion is at once removed by the post-mortem examination, which exhibits all the appearances not of an acute but of a chronic inflammation of the stomach, the tissues of which are found thickened and brawny, hard, and without any increase of vascularity. The abrupt even edges of the ulcers also are widely different from the softened ragged margins of those which are produced by any corrosive substance.—*London Med. Gaz.*

---

*Therapeutical action of Prussic Acid.*—In our last Number we related the results of some experiments, which have recently been made in the French hospitals with Prussic acid. The following contains a summary of M. Bequerel's researches:—

In diseases of the heart the use of Prussic acid was more injurious than useful, as it sometimes increased the distressing symptoms.

The anhydrous acid, prepared after the manner of Gea-Pessina, is the only one which should be employed medicinally: it keeps for a long time without being altered, and its action is uniform.

It should be given in a mixture of four ounces of water without sugar, and by spoonfuls. Uniformity in the strength of the doses is thus obtained.

When given in the dose of from 8 to 12 drops this acid acts locally, and with intermissions; but when carried to 16 or 20 drops, and continued for a certain time, its action is general and not intermittent: it is essentially hyposthenic.

In larger doses than those just mentioned Prussic acid is apt to occasion certain accidents, the chief symptoms of which consist in a violent excitement of the nervous and circulating systems.

It has no influence on the progress or symptoms of any disease; but in some nervous affections it may change the progress, intensity, and nature of the symptoms, without bringing about a complete cure.—*Lancet, from Gaz. Med. de Paris*, Jan. 13, 1840.

---

*Aneurism of the Aorta, with and without Bruit de Soufflet.*—Dr. Corrigan said that he exhibited these specimens together, because they agreed with one another in their pathological characters, with one single but most important exception. Both were examples of aneurisms of the ascending aorta; one of them had been taken from the body of a woman named Hamilton, the other from the body of a man named Dunn. They agreed in their size, situation, pathology, and even in their diagnostic signs, with this exception, that in the case of Dunn the bruit de soufflet was never absent, while in that of Hamilton it was never present. In the case of Dunn the aneurism involved the mouth of the aorta; in the case of Hamilton it did not, there being a portion of the vessel, about an inch and a half from its mouth, perfectly free from disease. In Dunn's case, too, the heart was very large; in Hamilton, it was below the natural size. In Hamilton's case the valves and the commencement of the aorta being sound, and the action of the heart weak, there was no vibratory motion communicated to the parietes to give rise to bruit de soufflet; but in Dunn's case there was a flaccid state of the heart, with disease of the aortic valves. Dr. Corrigan thought that, as a general rule, bruit de soufflet would be be found in all cases where the aortic valves were diseased, and that it would be absent where they were sound.—*Dublin Journal of Med. Science.*

---

*Œdema of the Larynx.*—Mr. Adams also exhibited a specimen of œdema of the larynx, taken from the body of a man, who, for twelve months previous to his death, had suffered from œdema of the lower extremities; about a week since, the scrotum and the penis became œdematous, the eyelids began to swell, and he got symptoms of effusion into the chest. The day before his death, he mas attacked with swelling of his neck, which became quite cilindrical, and respiration was greatly impeded. On the following morning, while endeavouring to swallow his breakfast, he fell back suddenly and expired. The cause of sudden death was found to be seated in the larynx; the submucous tissue about the glottis was distended with a yellowish serous effusion. It afforded a good specimen of the serous effusion of Bayle.

*Dublin Journal.*

---

*Hernia on the Left Lung following a wound of the Thorax, cured by pressure with a Truss.*—P. Valade, æt. 29, admitted into the hospital of La Charité, on the 21st July, 1839, under the care of M. Velpeau, on account of a small elastic swelling which had then existed for some time upon the front of the left breast. This patient, who has lately served in the army which occupies Algiers, stated that several months since he was wounded in a duel, the point of his antagonist's sword entering the left breast a little above and to the side of the nipple; he lost, at the time, a very large quantity of blood, and, being taken to the military hospital, remained under treatment for some weeks. The wound, which was of very small dimensions externally, soon healed, but not long after his discharge from the hospital, having quitted the service, and resumed his former occupation as a carpenter, a swelling begun to make its appearance underneath the cicatrix, and has gradually increased in its size up to the present time. The tumour only protrudes when he coughs, or makes any violent muscular exertion, when it is immediately puffed up to the size of a large egg. There can be no doubt that the lung had become adherent to the internal surface of the cicatrix, since it is impossible to effect the total reduction of the protrusion.

*July* 24.—The principal portion of the protruded lung has been reduced, and a truss has been applied with the intention of preventing its egress.

30.—The hernia still protrudes when the truss is removed, but it does not appear to be as large as before.

*Aug.* 11.—The employment of the truss appears to have answered the intentions with which it was had recourse to, since, when the pressure is removed, the hernia no longer protrudes.

Discharged cured.

In his clinical lecture upon this case, M. Velpeau stated that he had seen several instances of hernia of the lung occurring independently of any wound of the chest, and alluded to one, in particular, which existed in a young woman, where the protrusion had taken place in the subclavian region of the neck, and mounted up nearly as high as the border of the thyroid cartilage.—*Lancet.*

---

*Arabic mode of treating the Venereal Disease.*—This mode of treatment was communicated more than 150 years ago to the medical officers of the Marseilles' Hospital, by a Spanish apothecary, and is still employed there in all cases of virulent syphilis which resist the ordinary methods. The Arabic mode consists in the use of pills, of an opiate, sudorific drinks, and a peculiar regimen, called the *dry diet.* The pills are thus composed :—

Metallic mercury, Deuto-chloride of mercury, of each, ½ ʒ ;

Senna, Agaric, Root of Pyrethrum, of each, one ʒ ;

Honey, q. s.

The vegetable substances are reduced to powder; the metallic mercury is carefully rubbed up with the deuto-chloride, until the globules have completely disappeared, and the honey is then added, to make a mass, which is divided into six-grain pills. Two are given daily. The opiate is thus composed :—

Sarsaparilla, 5 ounces ;
Squine, 3 ounces ;
Roasted nut-shells, 1 ounce ;
Allspice, 1 drachm ;
Honey, q. s.

Two to four drachms are given night and morning.

The sudorific drink is made with sarsaparilla and squine. The patient is not allowed to touch any other fluid, but takes one or two quarts of this in the twenty-four hours. The diet is truly a *dry* one. Biscuits, raisins, nuts, dried figs, and roasted almonds ; no other food is allowed. The following order is prescribed for the administration of the remedies : a pill every morning, with a glass of the tisan ; an hour afterwards, the opiate with another glass; in the evening the same routine.

The duration of this treatment varies from thirty to fifty days. The enormous quantity (thirty-six grains) of corrosive sublimate contained in the pills might render us apprehensive of some accidents, if we were not aware that its properties were destroyed by being rubbed up with the metallic mercury. It has never been known to produce any unpleasant effects. However extraordinary this mode of treatment may appear to be, M. Payen, whose account of it we transcribe, assures us that it is regarded, at the Marseilles' Hospital as the most sure and efficacious in all cases where the constitution is deeply affected ; whenever ill-conditioned ulcers exist in the throat, with caries of the bones, &c.; and particularly whenever the ordinary treatment of syphilis has failed.—*London Lancet, from L'Experiènce,* No. 129, 1839.

---

*Salivation produced by Iodide of Potassium.*—At a meeting of the Medical Society of London, December 16, 1839, Mr. Crisp, in reference to the discussion at the last meeting, remarked that he had a case under his care, in which most profuse salivation was produced by the iodide of potassium. The patient, a woman, had taken the medicine for two or three months, for the removal of some venereal excrescences, in the neighbourhood of the labia. At the end of the time mentioned, salivation set in, and had continued up to the present time, a period of nearly five years; the quantity of saliva secreted, during the twenty-four hours, was about a quart. It was remarkable, that, on one or two occasions, the secretion had been arrested for a short time, from some unknown cause, and she then immediately experienced an unpleasant feeling in the neighbourhood of the disease, and this continued until the salivation returned. He had tried various remedies, acetate of lead, sulphur, astringents, and small doses of hydrargyrum cum creta among the others, without effect. He had seen another case in which the salivation had resulted from the use of the iodide of potassium; in this case there was not the peculiar fœtor of the breath which accompanied mercurial salivation. In these cases no mercury had been employed, previous to the use of the iodide of potassium. In the first case, small doses had been used previous to the potash, but no salivation had been produced.

